# Efficacy of physical exercise on the physical ability, cardiac function and cardiopulmonary fitness of patients with atrial fibrillation: a systematic review and meta-analysis

**DOI:** 10.3389/fcvm.2024.1352643

**Published:** 2024-02-23

**Authors:** Chenyan Yang, Qian Wu, Qianyu Lv, Xinzheng Hou, Xuejiao Ye, Yingtian Yang, Lanlan Li, Wenxi Zuo, Shihan Wang

**Affiliations:** ^1^ Guang’anmen Hospital, Chinese Academy of Chinese Medical Sciences, Beijing, China; ^2^ Guang'anmen Hospital, Beijing University of Chinese Medicine, Beijing, China

**Keywords:** atrial fibrillation, physical exercise, cardiopulmonary fitness, cardiac function, physical ability

## Abstract

**Objective:**

It is advised that patients engage in physical activity to enhance their quality of life and achieve better results. The purpose of the current study was to measure the efficacy of exercise on the physical ability, cardiac function and cardiopulmonary fitness of patients with AF.

**Method:**

A comprehensive systematic literature search was performed in PubMed, Embase, and Web of Science from 1991 to 2023 for RCTs comparing physical exercise combined with AF routine treatments to routine treatments alone. The meta-analysis was conducted following PRISMA guidelines. Our main outcomes were physical ability (measured by the 6-min walk test, 6MWT), cardiac function (measured by left ventricular ejection fraction, LVEF) and cardiopulmonary fitness (measured by peak oxygen uptake and resting heart rate). Quality assessments were conducted using the Cochrane Collaboration tool.

**Results:**

Thirteen trials involving 672 patients met the criteria for analysis. The results showed that physical exercise increased physical ability by improving the 6MWT (m) performance (MD = 96.99, 95% CI: 25.55–168.43; *Z* = 2.66; *p* = 0.008); and enhanced peak VO2 (ml/kg per min) (MD = 4.85, 95% CI: 1.55–8.14; *Z* = 2.89; *p* = 0.004) while reducing resting heart rate (beats per minute, bpm) (MD = −6.14, 95% CI: −11.30 to −0.98; *Z* = 2.33; *p* = 0.02). However, the results showed that regular exercise could improve LVEF (%) inpatients clinically, which had no statistic difference between experimental and control group (MD = 1.49, 95% CI: −0.25–3.24; *Z* = 1.68; *p* = 0.09).

**Conclusion:**

Our meta-analysis shows that physical exercise is an effective intervention to improve the exercise ability and cardiopulmonary fitness for AF patients. Meanwhile, we also do not exclude the positive effect of exercise on the improvement of cardiac function (LVEF) in patients with AF. To this end, doctors should consider the positive impact of exercise on patients and give advice on exercise limits in practical clinical practice.

## Introduction

The most prevalent prolonged heart arrhythmia, atrial fibrillation (AF), is linked to significant morbidity, mortality, and healthcare utilization (1). The incidence and prevalence of atrial fibrillation (AF) have reached the level of a cardiovascular disease (CVD) epidemic in the twenty-first century due to rising average worldwide life expectancy and extended survival with chronic diseases (2). Preventing atrial fibrillation (AF) and its associated consequences remains problematic despite extensive research efforts (3). The aging of our population and the rise in obesity are two factors that are strongly linked to and may even be the cause of AF, and they may be contributing to its incidence. Modifiable cardiovascular risk factors, including obesity, hypertension (HTN), diabetes mellitus, obstructive sleep apnea (OSA), alcohol consumption, smoking, and sedentary lifestyles, have been proposed as possible contributors to the development and progression of AF (4). Significant decreases in the burden of AF were observed with effective weight loss and enhanced physical activity (1).

As a result, studying the connection between exercise and AF is a highly worthwhile endeavor. Physical exercise can effectively assist in the therapy of various types of cardiovascular diseases, and the potential benefits of moderate regular physical exercise in reducing the risk of AF are considerable (5). Several studies show that regular physical activity positively benefits AF prevention (3). Participation in an exercise-based intervention over 6 months reduced arrhythmia recurrence and improved symptom severity among patients with AF (6). Research has demonstrated that consistent physical activity has an inverse and independent relationship with the advancement of clinical AF (7). We aim to see if exercise can help atrial fibrillation patients live longer and have better prognoses.

Fewer types of AF patients were included in the study (8, 9), and the effect of exercise on cardiac function in patients with AF is unclear despite previous meta-analyses in this area. After searching relevant experiments, we found some controversy about whether exercise can improve the LVEF in patients with atrial fibrillation. While some randomized trials (10) found that exercise enhanced left cardiac function, others (6, 11) found no significant changes. Thus, we gathered these data for analysis. We also need to revise the results in light of the new study reports.

Thus, this meta-analysis aims to investigate areas that remain unclear and update the existing findings. Our responsibility is to clarify how exercise affects an AF patient's physical capacity, heart function, and cardiopulmonary fitness. Furthermore, we broadened the scope of the research participants to provide a more thorough framework for the clinic.

## Methods

This present meta-analysis was conducted following by the Preferred Reporting Items for Systematic Reviews and Meta-Analyses (PRISMA) guidelines. The data used in this study are all secondary and do not require ethical approval.

### Search strategy

A systematic literature search was conducted in five electronic databases, including PubMed, Embase and Web of Science from 1991 to 2023 for RCTs studying the effects of physical exercise on the physical ability, cardiac function and cardiopulmonary fitness of patients with AF. Keywords and their medical subject headings (MeSH) or Embase subject headings (EMTREE) were used for the search strategy. Table 1 shows the details of the search strategy.

**Table 1:** Details of the search strategy.

No.	Query
Pubmed search strategy	
1	“Atrial Fibrillation”[Mesh]
2	(((((Atrial Fibrillation[MeSH Major Topic]) OR (Atrial flutter [MeSH Major Topic])) OR (Atrial Fibrillation [Title/Abstract])) OR (Atrial flutter[Title/Abstract])) OR (Auricular Fibrillation [Title/Abstract])) OR (Auricular flutter[Title/Abstract])
3	#1 OR #2
4	“Exercise”[Mesh]
5	(((((((Exercise[MeSH Major Topic]) OR (Activity[MeSH Major Topic])) OR (Exercise[Title/Abstract])) OR (Activity[Title/Abstract])) OR (Physical Exercise[Title/Abstract])) OR (Physical Activity[Title/Abstract])) OR (Aerobic Exercise[Title/Abstract])) OR (Exercise Training [Title/Abstract])
6	#4 OR #5
7	“Randomized Controlled Trial” [Publication Type]
8	#3 AND #6 AND #7
No.	Query
Emabse search strategy	
#1	“atrial fibrillation”/exp
#2	“atrium fibrillation”:ab,ti OR “auricular fibrilation”:ab,ti OR “auricular fibrillation”:ab,ti OR “cardiac atrial fibrillation”:ab,ti OR “cardiac atrium fibrillation”:ab,ti OR “fibrillation, heart atrium”:ab,ti OR “heart atrial fibrillation”:ab,ti OR “heart atrium fibrillation”:ab,ti OR “heart fibrillation atrium”:ab,ti OR “non-valvular atrial fibrillation”:ab,ti OR “nonvalvular atrial fibrillation”:ab,ti OR “atrial fibrillation”:ab,ti
#3	#1 OR #2
#4	“exercise”/exp
#5	“biometric exercise”:ab,ti OR “effort”:ab,ti OR “exercise capacity”:ab,ti OR “exercise performance”:ab,ti OR “exercise training”:ab,ti OR “exertion”:ab,ti OR “fitness training”:ab,ti OR “fitness workout”:ab,ti OR “physical conditioning, human”:ab,ti OR “physical effort”:ab,ti OR “physical exercise”:ab,ti OR “physical exertion”:ab,ti OR “physical work-out”:ab,ti OR “physical workout”:ab,ti OR “exercise”:ab,ti
#6	#4 OR #5
#7	“randomized controlled trial”/exp
#8	“controlled trial, randomized”:ab,ti OR “randomised controlled study”:ab,ti OR “randomised controlled trial”:ab,ti OR “randomized controlled study”:ab,ti OR “trial, randomized controlled”:ab,ti OR “randomized controlled trial”:ab,ti
#9	#7 OR #8
#10	#3 AND #6 AND #9
No.	Search query
Web of science search strategy	
#1	Exercise (Topic) OR Activity (Topic) OR Physical Exercise (Topic) OR Physical Activity (Topic) OR Aerobic Exercise (Topic) OR Exercise Training (Topic)
#2	Atrial Fibrillation (Topic) OR Atrial flutter (Topic) OR Auricular Fibrillation (Topic) OR Auricular flutter (Topic)
#3	Randomized Controlled Trial (Topic)
#4	#1 AND #2 AND #3

### Inclusion criteria

Eligible studies were identified if they met all the following criteria:

(1) Population: adults with AF (aged 18 and over with no upper age limit), including paroxysmal AF patients, persistent AF patients, permanent AF patients, chronic AF (a longstanding chaotic and irregular atrial arrhythmia) patients, patients with atrial fibrillation who underwent the radiofrequency ablation and permanent AF in HF patients.
(2) Intervention: studies reporting the effects of physical ability, cardiac function and cardiopulmonary fitness of AF patients.
(3) Comparison: treatment as usual or daily exercise (not participated in any organized sports programs).
(4) Outcome(s): physical ability measured by the 6MWT, cardiac function measured by LVEF and cardiopulmonary fitness measured by peak oxygen uptake (peak VO2) and resting heart rate.
(5) Study type: RCT.

### Exclusion criteria

(1) Animal studies, case reports, reviews, abstracts, conference proceedings and editorials.
(2) Non-RCTs.
(3) Studies without sufficient data to calculate WMD.

### Data extraction

WPS Office was used to set up the data extraction table. The main components of the extracted information were classified as:

(1) Publication information: first author and publication year.
(2) General characteristics of patients: sample size, gender and age.
(3) Details of intervention and control therapy: training period, session duration, training mode and number of sessions per week.
(4) Details of outcomes.
(5) Bias risk assessment information: quality of included studies.

### Statistical analyses

Review Manager 5.4 was used for all statistical analyses. We used the WMD with the corresponding 95% confidence intervals (CI) as the effect size (ES). If the data (including the sample size, mean value and standard deviation) extracted from the RCTs met the needs of the analyses, the analyses were conducted directly (12). If the data (including the standard error, 95% CI or *p*-values) in the RCTs could not be used now for analyses, they were analyzed after data conversion (13–15). Cochran's *Q*-test and I2 test were used to evaluate heterogeneity. A *p*-value <0.1 or I2 value >50% indicated significant heterogeneity between RCTs, and the random-effect model was used. Otherwise, a fixed-effect model was used.

### Assessment of the risk of bias

The Cochrane Collaboration tool was used to assess the risk of bias. Two authors independently extracted data and assessed the quality of the included studies. The data were recorded on a special data form. The differences between data extraction and quality evaluation were determined through discussion.

## Results

### Search results

**Figure 1:**
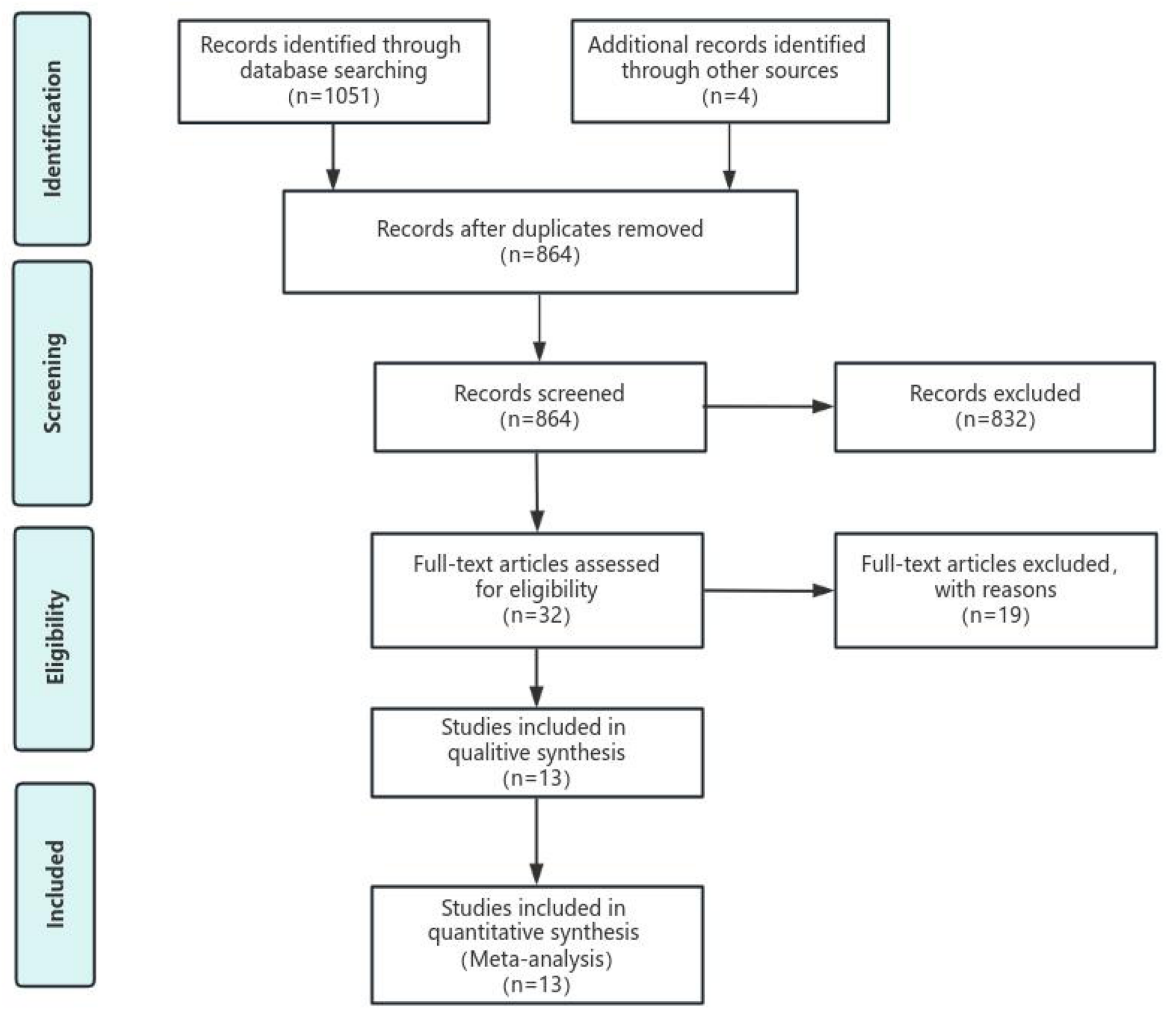
Flow diagram of the search procedure.

**Figure 2:**
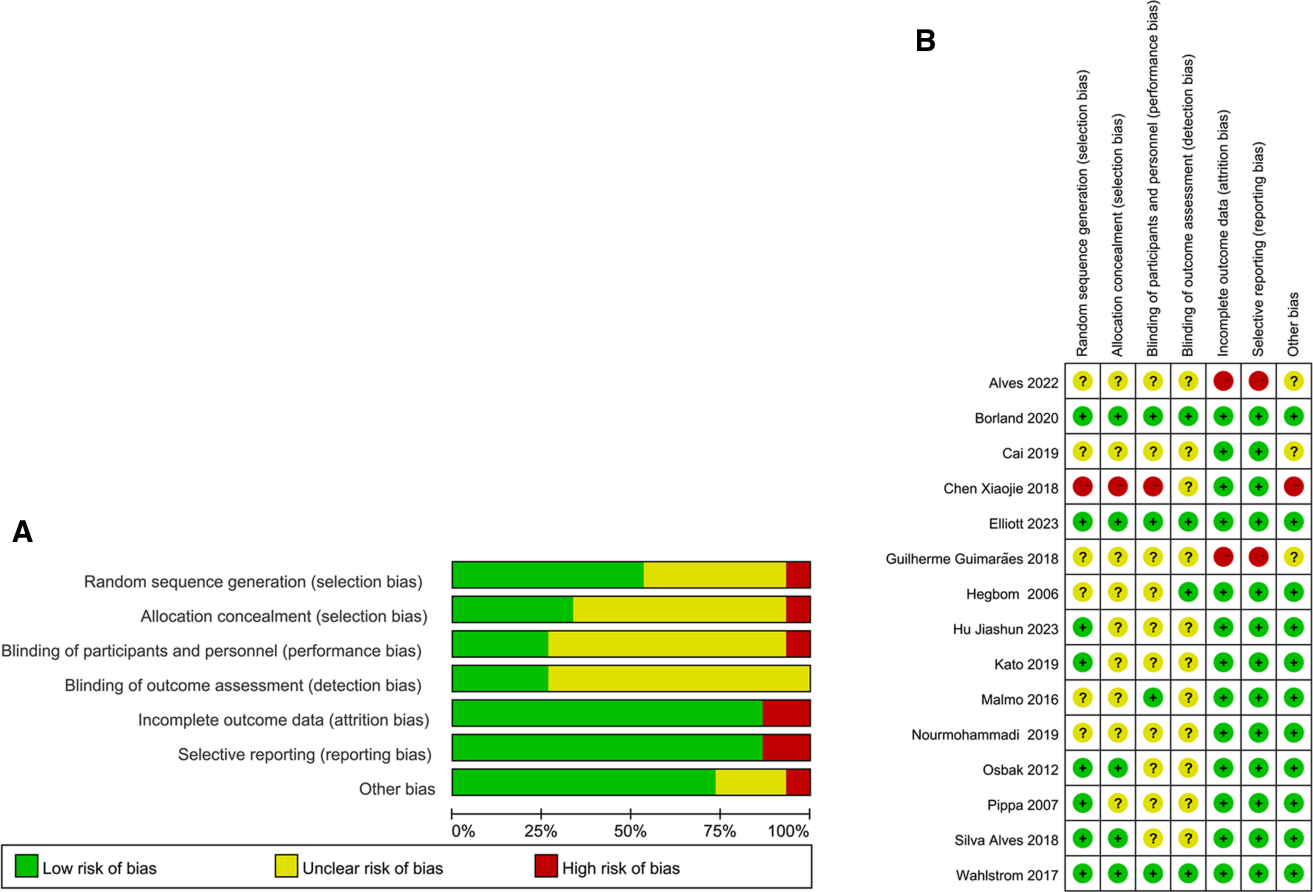
(**A**) The risk of bias. A review of the authors’ judgments about each risk of bias items are presented as percentages across all of the included studies. The quality of the selected is assessed according to the Cochrane criteria. (**B**) The risk of bias summary. A review of the authors’ judgments about the risk of bias are included in each study.

Initially, 1,070 potential studies were retrieved. After deleting duplicates, 864 studies were screened. After scanning the titles and abstracts, the inconsistencies were eliminated, and 32 papers remained. After carefully reading the full text using pre-established inclusion and exclusion criteria, 19 articles were excluded, and the remaining 13 were included in the meta-analysis. The flow chart of the search process is shown in Figure 1. The bias condition of selected studies is shown in Figures 2A,B.

### Trial and patient characteristics

The 13 RCTs included 672 patients (335 allocated to experimental groups and 337 allocated to control groups). Four RCT exercise interventions were aerobic, six were aerobic combined with anaerobic, one was Qigong and one was Yoga. The details of all included studies are shown in Table 2.

**Table 2:** Data extraction of the selected articles.

	Study	Country	Inclusion criteria	Case (*n*)		Male (*n*)	Training mode	Control measures	Primary outcomes
				EG	CG				
1	(16)	China	Patients with atrial fibrillation treated by catheter ablation	29	27	44	Aerobics	Usual care	Cardiac function (peak VO2) Exercise capacity (6MWT) QoL (SF-36) Resting HR BP
2	(17)	Norway	Patients with chronic AF	13	15	26	Aerobic exercise and muscle strengthening	Usual care	Heart rate variability (HF) Exercise capacity (CW) QOL (SF-36)
3	(18)	Brazil	Permanent AF in HF patients	12	12	Not clear	Aerobics	Maintain habitual activities	BNP ANP VO2 resting and maximum heart rate
4	(19)	Brazil	AFp associated with HF	13	13	Not clear	Combination	Usual activities	LAD LVEF VE/VCO2
5	(20)	America	Nonpermanent AF patients	26	25	22	Aerobics	Continued their previous exercise habits.	Time in AF Vo2 peak AF Symptoms and QoL (SF-36)
6	(21)	Norway	Patients with paroxysmal atrial fibrillation	33	36	42	Yoga	Not to perform any yoga	QoL Haemodynamic assessments (BP, HR)
7	(10)	Japan	Patients treated with catheter ablation for persistent atrial fibrillation	28	31	47	Combination	Usual care	Exercise capacity and physical function (6MWD, peak VO2) Cardiac structure and function (LVEF%)
8	(22)	Denmark	Adults with permanent atrial fibrillation	24	23	35	Combination	Usual care	Muscle strength Exercise capacity and 6-min walk test. Heart rate and blood pressure QoL
9	(23)	Brazil	Patients with HFAF	13	13	26	Combination	Untrained	Resting HR, HR recovery Cardiopulmonary exercise (peak vo2, VE/VCO2) Echocardiography QoL
10	(6)	Australia	Patients with symptomatic paroxysmal or persistent AF	51	46	69	Combination	Usual care	AF Monitoring AF Symptom Severity Cardiopulmonary Exercise Testing (VO2 peak)
11	(11)	Italy	AF patients	22	21	30	Qi gong	Usual care	6-minute walk ejection fraction total cholesterol, high-density lipoprotein, homocysteine
12	(24)	Sweden	Permanent AF patients	46	50	68	Combination	Positive effects of physical activity on prescription	Exercise capacity Muscular endurance tests Physical activity level QoL Cardiac function (EF)
13	(25)	Iran	Chronic AF patients	25	25	23	Aerobic exercises	Not participate in any organized sports programs	QoL

### Effects of physical exercise on exercise ability in patients with AF

**Figure 3:**
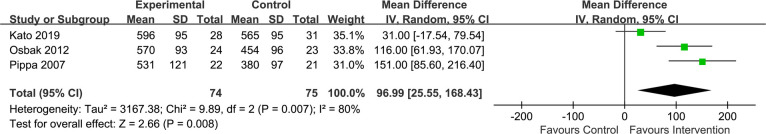
Forest plot shows the ES of physical on 6MWT in AF patients.

The effect of physical exercise on exercise ability was measured by the 6MWT. Three RCTs (10, 11, 22) reported the results of 6MWTs, which had statistic difference between experimental and control group (MD = 96.99, 95% CI: 25.55–168.43; *Z* = 2.66; *p* = 0.008) (Figure 3).

### Effects of physical exercise on cardiac function in patients with AF

**Figure 4:**
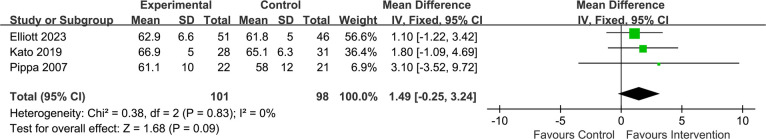
Forest plot shows the ES of physical exercise on LVEF in AF patients.

The effect of physical exercise on cardiac function was measured by LVEF. Three RCTs (6, 10, 11) reported the results of LVEF, which had no statistic difference between experimental and control group (MD = 1.49, 95% CI: −0.25–3.24; *Z* = 1.68; *p* = 0.09) (Figure 4).

### Effects of physical exercise on cardiopulmonary fitness in patients with AF

**Figure 5:**
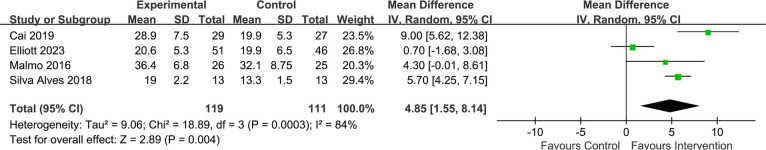
Forest plot shows the ES of physical exercise on peak vo2 in AF patients.

**Figure 6:**
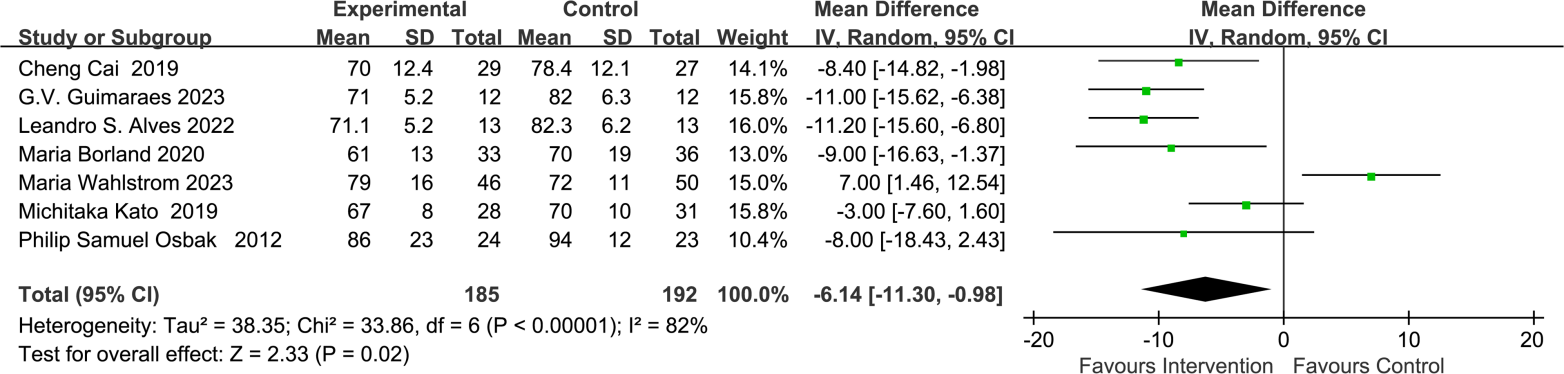
Forest plot shows the ES of physical exercise on resting heart rates in AF patients.

The effect of physical exercise on cardiopulmonary fitness was measured by the peak VO2 and resting heart rate. Four RCTs (6, 16, 20, 23) reported peak VO2 values, which had statistical differences between the experimental and control groups (MD = 4.85, 95% CI: 1.55–8.14; *Z* = 2.89; *p* = 0.004) (Figure 5). Seven RCTs (10, 16, 18, 21–24) reported resting heart rates, which had statistic difference between experimental and control group (MD = −6.14, 95% CI: −11.30 to −0.98; *Z* = 2.33; *p* = 0.02) (Figure 6).

### Publication bias

**Figure 7:**
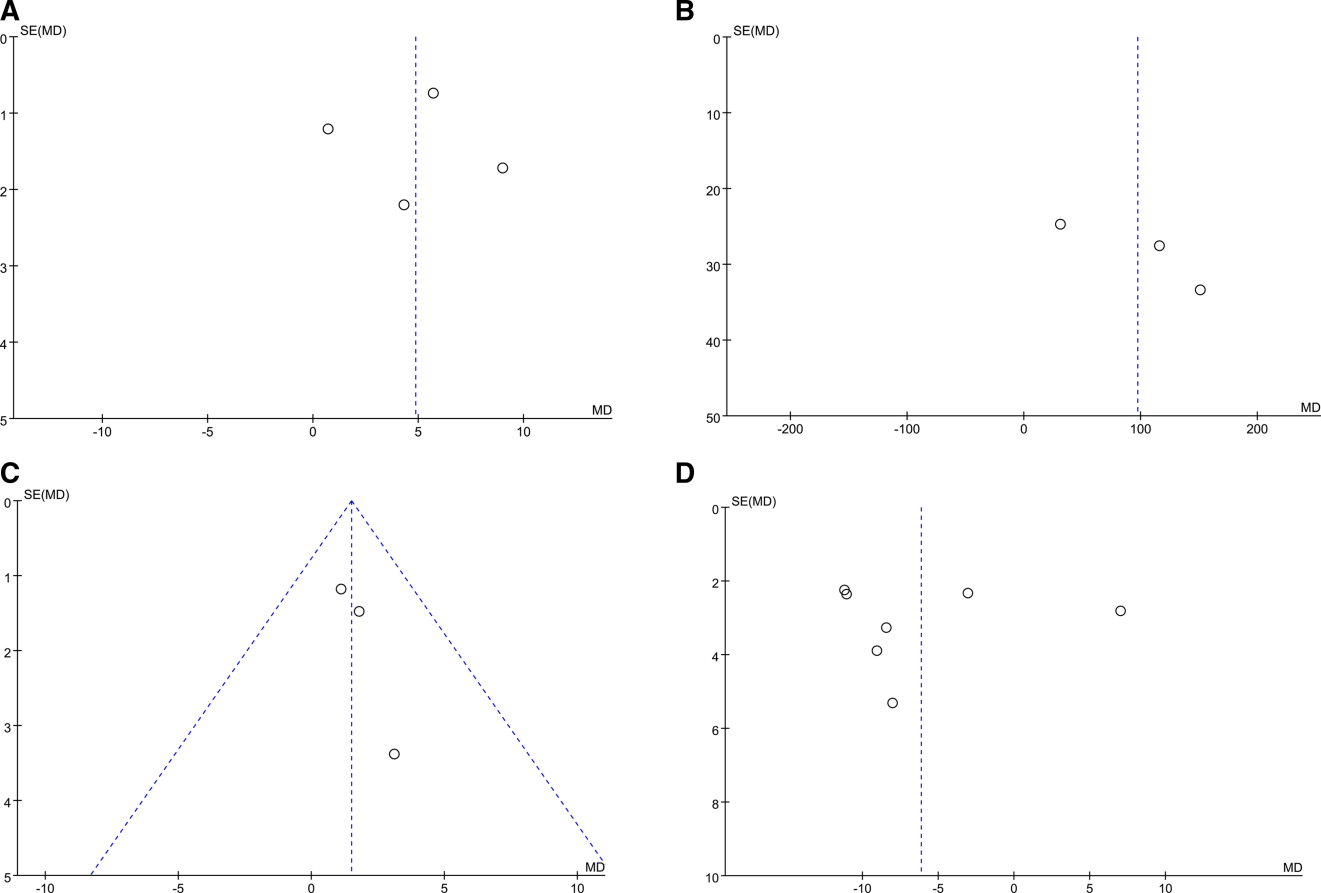
Funnel plots shows the publication bias of physical exercise on peak vo2 (**A**), 6MWT (**B**), LVEF (**C**), resting HR (**D**) in AF patients.

Review Manager 5.4 was used to draw the funnel plots to analyze the data related to the individual outcome indicators. From the figure, the publication bias of each group is not apparent, and the figure roughly shows the symmetry of the centreline concentrated in the upper part (Figure 7).

## Discussion

From all the results above, we found that physical exercise positively affected the exercise ability and cardiopulmonary fitness in patients with AF. Statistics did not indicate the difference between exercise and the control group of cardiac function.

Compared with the previous analysis (8, 9), our study included more types of patients with AF, such as patients with AF after radiofrequency ablation and patients with heart failure with AF (10, 16, 18, 19, 23). The reason why we do so is mainly to consider that the clinical condition of some AF patients is more complex. Therefore, we included as many patients with various types of AF as possible. This has important implications for our patient education in the clinic. Secondly, we focused on the efficacy of exercise on the physical ability, cardiac function and cardiopulmonary fitness of patients with AF. What we included more than the previous meta was the outcome “LVEF”, which helped to respond to the controversy about the aspect. This reminded us that we could pay attention to the LVEF of AF patients during exercise. In addition, our study was an updated analysis, including statistics according to inclusion criteria from 1991 to 2023. We are committed to presenting the latest research results to readers to guide clinical and life better.

There were similar reports in the previous analysis for the results of improvement on 6MWT and peak VO2 (8, 9). This suggests that we could improve patients' physical quality and exercise ability with AF through effective and appropriate exercise methods. There appeared to be no differences in outcomes between exercise at high and lower intensity (26).

Resting heart rate is a relatively simple and direct indicator of understanding heart function. The decrease in resting heart rate after exercise intervention also suggests that exercise benefits cardio respiratory fitness in patients with AF. At the same time, resting heart rate is often used to measure the patient's exercise limit (70%–90% of maximal heart rate usually), which is very beneficial for clinical patient exercise monitoring (17).

We were skeptical about whether exercise could improve the ejection fraction in patients with AF. Even if the results of our analysis showed no statistically significant difference between the two groups, we could believe that exercise in patients with AF is clinically significant. After all, several studies have shown that patients' ejection fraction increases significantly after exercise (6, 10, 11). The number of sample sizes and the study subjects' baseline characteristics were considered relevant factors. We hope that more studies will be done to support the relationship between exercise and ejection fraction in patients with AF.

As for the exercise duration, the 2018 Physical Activity Guidelines Advisory Committee Report recommends 150 min/per week of moderate-intensity or 75 min/per week of vigorous-intensity aerobic exercise for all adults because this exercise volume improves cardiovascular health (1). American Heart Association recommends moderate-intensity aerobic exercise for about 60 min every day for several months to reduce the risk of cardiovascular disease (8). Most importantly, this higher risk of AF has been observed only with exercise doses that far exceed recommendations of the Physical Activity Guidelines Advisory Committee Report and are therefore achieved by only <1% of Americans (1). But in real life, only a subset of patients can achieve the above standards due to the differences in personal physical quality or other reasons. We recommend that patients choose exercise limitation based on the degree of physical fatigue, which is consistent with our clinical patient education.

## Strengths and limitations

We believe this is the latest analysis of AF patients exercise, which has a broad range population, including paroxysmal AF patients, permanent AF patients, chronic AF patients, patients with atrial fibrillation who underwent radiofrequency ablation, and permanent AF in HF patients. At the same time, we conducted statistical analysis for previous studies with inconsistent results on LVEF to guide clinical practice better. However, we recognize that there are some limitations to this study. First, the large number of types of patients with AF may lead to differences between different populations. Second, the small sample size in multiple studies may make the results less representative. Otherwise, individual studies have differences in baseline patient characteristics, which may affect the final study results. Thirdly, our study did not focus on the effect of exercise on AF recurrence, which also plays an essential role in clinical guidance. In the future, we expect to collect more studies with larger sample sizes for analysis and group them according to different baseline characteristics. And we hope that more researchers will work to study the effects of exercise on the recurrence of AF.

## Conclusions

Our meta-analysis shows that physical exercise is an effective intervention to improve AF patients' exercise ability and cardiopulmonary fitness. Meanwhile, we also do not exclude exercise's positive effect on improving cardiac function (LVEF) in patients with AF. To this end, doctors should consider the positive impact of exercise on patients and advise on exercise limits in practical clinical practice. We should continue to expand the accumulation amount of related studies in the future to help patients with different types of AF better.

## Data Availability

The original contributions presented in the study are included in the article/Supplementary Material, further inquiries can be directed to the corresponding author.
